# Benchmarking of Contactless Heart Rate Measurement Systems in ARM-Based Embedded Platforms

**DOI:** 10.3390/s23073507

**Published:** 2023-03-27

**Authors:** Andrea Manni, Andrea Caroppo, Gabriele Rescio, Pietro Siciliano, Alessandro Leone

**Affiliations:** National Research Council of Italy, Institute for Microelectronics and Microsystems, 73100 Lecce, Italy

**Keywords:** remote PPG, contactless monitoring, heart rate, ARM-based embedded platforms, benchmark analysis

## Abstract

Heart rate monitoring is especially important for aging individuals because it is associated with longevity and cardiovascular risk. Typically, this vital parameter can be measured using wearable sensors, which are widely available commercially. However, wearable sensors have some disadvantages in terms of acceptability, especially when used by elderly people. Thus, contactless solutions have increasingly attracted the scientific community in recent years. Camera-based photoplethysmography (also known as remote photoplethysmography) is an emerging method of contactless heart rate monitoring that uses a camera and a processing unit on the hardware side, and appropriate image processing methodologies on the software side. This paper describes the design and implementation of a novel pipeline for heart rate estimation using a commercial and low-cost camera as the input device. The pipeline’s performance was tested and compared on a desktop PC, a laptop, and three different ARM-based embedded platforms (Raspberry Pi 4, Odroid N2+, and Jetson Nano). The results showed that the designed and implemented pipeline achieved an average accuracy of about 96.7% for heart rate estimation, with very low variance (between 1.5% and 2.5%) across processing platforms, user distances from the camera, and frame resolutions. Furthermore, benchmark analysis showed that the Odroid N2+ platform was the most convenient in terms of CPU load, RAM usage, and average execution time of the algorithmic pipeline.

## 1. Introduction

Telemedicine is rapidly developing due to the availability of commercial measurement instruments, which are becoming more affordable and user-friendly. It involves exchanging medical information from one location to another using electronic communication, which can improve patient health status [1,2]. Telemedicine services include patient monitoring at remote locations using devices that collect and send patient data to home health agencies or diagnostic testing facilities. The information might include specific vital signs for homebound patients, such as heart rate (HR), heart rate variability (HRV), and ECG information. Previous parameters were typically measured using traditional and commercial contact-based methods, such as wristbands and smartwatches. Wearable sensors have the advantage of higher localization accuracy and tracking, but they are more uncomfortable in nature [3]. Moreover, wearable sensor-based monitoring demands end-users to remember to wear the devices as well as charge the devices regularly. This difficulty is accentuated with advancing age, making the use of wearable devices more difficult for the elderly. Contactless sensors (i.e., camera-based) are less intrusive and can monitor vital signs without causing any interference to an individual’s daily routines.

Recent literature shows that scientific approaches based on assessing color intensity variations within a specific color space are the most investigated. They are based on the remote plethysmography signal (rPPG) acquired by a vision sensor [4]. The principle of rPPG is simple: reflected light from certain regions of the skin is affected by the amount of blood under the skin, and this reflected light can be used to measure blood volume changes. The subtle changes in human skin color are invisible to the human eye but they can be evaluated using a commercial vision sensor.

**Figure 1:**
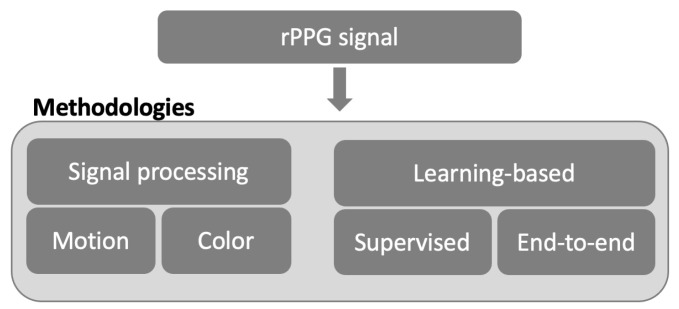
Classification of processing approaches for HR estimation through rPPG signals, using a stream of images as input.

Researchers have developed a variety of innovative rPPG methods to obtain HR, i.e., with inexpensive digital cameras [5,6,7,8]. Moreover, several review articles covering multiple elements of the non-contact monitoring of cardiac signals have extensively documented all of these advances [9,10,11]; some of them have even led to commercial solutions. Regarding the methodological aspects, two main categories for the estimation of HR (starting from the rPPG signal) have been investigated in the literature (a) signal processing techniques; (b) learning-based methodologies. The approaches of the first category can be defined as “unsupervised” and analyze either the motion present in the image (motion-based approaches) or the variation of the color intensity in a specific color space. On the other hand, learning-based approaches can be both supervised and focused on end-to-end architectures. Figure 1 illustrates the previously introduced categorization.

The following tasks are mostly carried out by the existing contactless HR estimation methods based on rPPG signals. Initially, a region of interest (ROI) is extracted. Next, a temporal signal is estimated after denoising, detrending, and normalization. Finally, the resulting signal is used to extract the rPPG signal, which is analyzed in the frequency domain and allows providing a quantification of the HR value. Several factors affect the correct measurement of HR from a pipeline structured according to the description above. These factors include the distance of the observed subject, the orientation of the face, the presence of motion artifacts, the age and skin tone of the subject, the ambient lighting conditions, and the selection of the ROI for signal extraction.

For example, in [12], the authors investigated the influence of distance on the HR estimation from a video recorded with a smartphone’s frontal camera. They evaluated the performance of six different algorithms to identify the best one for HR estimation. Their results demonstrated that the root-mean-square error (RMSE) and mean absolute error (MAE) increased as the camera distance increased. They identified the Plane Orthogonal to Skin (POS), green channel, and chrominance-based signal processing method (CHROM) as the best algorithms for estimating HR. The work described in [13] pointed out that the thickness of the skin is not uniform in all areas of the face, so the same diffused reflection information cannot be obtained in each area. Consequently, to see the effect of skin thickness on the accuracy of the rPPG algorithm, the authors experimented on 39 anatomically divided facial regions. The results showed that higher accuracy in HR measurement is obtained in the forehead and cheek areas of the subject. Moreover, in [14], the lighting conditions and camera shutter time on HR estimation using rPPG were investigated. This study concluded that for lighting conditions between 132–548 lux, a range of shutter times (5–32 ms) existed, for which a mean 3 BPM agreement between 90 and 100% was achieved.

Regarding the influence of skin tone, it is well known that dark skin, which contains higher amounts of melanin, fundamentally reduces the signal-to-noise ratio of all existing rPPG algorithms. Consequently, in [15], the authors presented a novel approach to mitigate bias for skin tone, inspired by the work of Kumar et al. [16]. The focus of this work was on understanding the unique physics that underlie inconsistencies in rPPG measurement. Using physics-rooted knowledge and camera noise analysis, the authors proposed modifications to existing rPPG denoising methods that use a similar weighted ROI philosophy as in [16]. Moreover, to overcome motion artifacts, Yu et al. [17] proposed a method of planar motion compensation and evidenced that the method could overcome motion artifacts and extract HR and breath rate (BR) even under high-intensity exercise by measuring pulses of 12 subjects before, during, and after cycling.

In the literature, recent trends include learning-based rPPG measurements, which offer the major benefit of detecting HR directly from video input and allowing the system to learn the rPPG mechanism from the beginning. Learning-based techniques can be divided into two main categories: supervised learning methods and end-to-end learning methods. For example, in [18], the authors described a novel deep HR methodology based on ’front-end’ and ’back-end’ machine learning (ML) strategy. Specifically, the back-end component is a fully connected neural network for HR estimation, whereas the front-end component learns independently from training video samples. The methodology was tested on two well-known literature datasets and achieved very low RMSE and acceptable processing times. Moreover, in [19], the authors proposed a ML approach that evaluated and compared the independent component analysis (ICA) method with two ML techniques in a controlled environment: the k-nearest neighbor (kNN) classifier and linear regression (LR).

Among the end-to-end learning methods, the first was introduced in [20]. Here, the authors introduced “DeepPhys”, a method able to extract HR and BR from a streaming video using a convolutional attention network (CAN) that enabled spatiotemporal visualization of the signals. DeepPhys was evaluated on three different literature datasets. The results showed very good performance when compared with the state-of-the-art approaches. Afterwards, to obtain a HR estimation in a contactless way, an unsupervised learning-based method called “SynRhythm” was developed [21]. The HR was estimated by extracting the blood volume pulse from a series of photos using two successive convolutional neural networks (CNNs).

However, these networks require large-scale labeled data for better generalization. Furthermore, for their operation, they require specific hardware resources that are not low in cost and, consequently, represent solutions that cannot be distributed on a large scale. Embedded systems are utilized in our everyday lives, from smartphones and tablets to computers, medical devices, and other electronic gadgets that provide high-computing capabilities. In the 1980s, Acorn Computers developed the first Advanced RISC Machine (ARM) processor at Cambridge University in England for commercial purposes. These ARM processors were further enhanced to provide high-performance and efficient power management without disrupting the system’s overall efficiency. Some of the main advantages of commercial ARM-based embedded platforms include affordability, as they do not require expensive production equipment. Compared to other processors, they are created and produced at a much lower cost. Additionally, ARM-based embedded platforms have lower power consumption and are composed of simple circuits, making them compact and suitable for use in smaller devices. Finally, ARM performs a single operation at a time, allowing for faster processing with lower latency and quicker response time.

Almost all literature and commercial solutions designed and implemented for contactless HR estimation are based on PC or laptop use; thus, from a scientific point of view, it would be interesting to design an algorithmic pipeline that cuts across multiple hardware architectures. To the best of our knowledge, only a few publications [22,23,24] have proposed algorithmic pipelines capable of estimating HR in a contactless mode and using single-board computers (i.e., Raspberry Pi or Jetson Nano).

The main contributions of this paper are essentially two-fold. First, we describe the design and implementation of an algorithmic pipeline for HR estimation of the observed user. The algorithmic steps of the pipeline are designed and optimized for proper operation on a variety of HW architectures. Second, we compare the performance of the pipeline in terms of measurement accuracy and computational load by testing its operation on three different ARM-based embedded platforms running a Linux operating system, on a stationary PC running Microsoft Windows, and on an Apple laptop with the macOS operating system. Our proposed solution allows for the use of any low-cost commercial camera, including the built-in webcam on a laptop, for input data acquisition and a variety of single-board computers for data processing.

The remainder of this paper is structured as follows. Section 2 explains our proposed solution for HR estimation, providing details on both hardware (specifications inherent in the commercial vision sensor used and all processing units compared) and software aspects (detailing each step integrated into the algorithmic pipeline). The results are presented in Section 3. Finally, Section 4 shows both our conclusions and discussions on ideas for future work.

## 2. Materials and Methods

This section begins with an overview of the hardware involved and tested for the implementation of the proposed pipeline. Following this, a detailed description of the algorithmic steps that are designed and implemented for contactless HR estimation is given.

### 2.1. Vision Sensor

**Figure 2:**
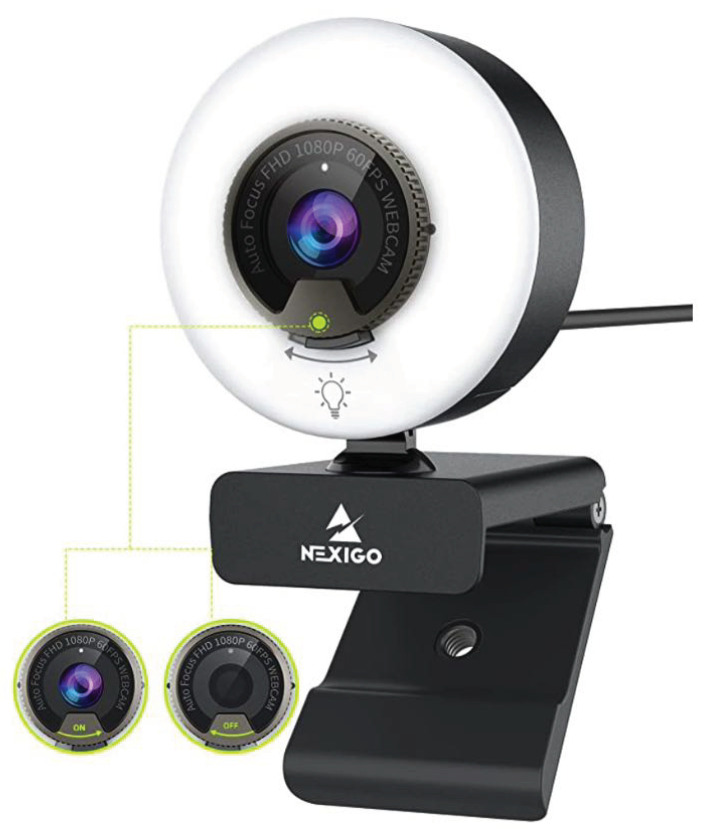
Nexigo webcam (model N960E).

On the hardware side, the current version of the proposed pipeline integrates a camera named N960E (Figure 2) and is commercially distributed by Nexigo [25]. The webcam has a 1920 × 1080 p high-definition resolution with a refresh rate of up to 60 frames per second (fps), a weight of 240 g, and small dimensions (5.99 × 4.52 × 8.71 cm). The advanced autofocus helps capture accurate and life-like videos/images as accurately as possible. In addition, the built-in ring light offers 3 lighting modes and continuously adjustable brightness. The latter feature makes the use of the webcam advantageous in application contexts (i.e., where the influence and variation of ambient brightness affect the proper functioning of the proposed application). Finally, it is equipped with a 1.5 m USB connection cable that enables its use even at a distance from the elaboration unit.

### 2.2. Elaboration Units

**Figure 3:**
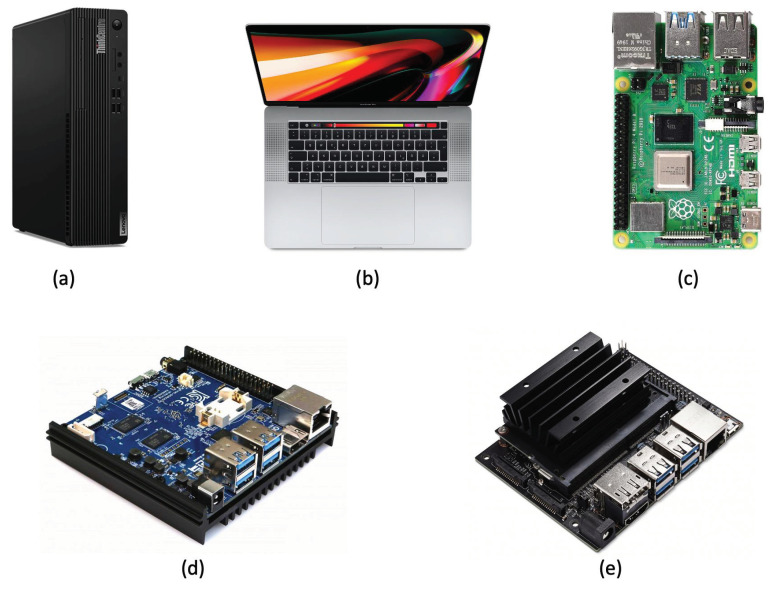
Elaboration units (Lenovo ThinkCentre (**a**), Apple MacBook Pro (**b**), Raspberry Pi 4 Model B (**c**), Odroid N2+ (**d**), and Jetson Nano (**e**)) for processing and estimation of the HR.

A desktop PC, a laptop, and three embedded architectures were used for the implementation of the proposed framework for comparison. The embedded architectures used were: Raspberry Pi 4 Model B, Odroid N2+, and Jetson Nano. The employed processing units are depicted in Figure 3. The ambient sensor was connected to these units via a USB while the algorithms for acquisition and processing were integrated into the units. The characteristics of each elaboration unit involved are shown below.

The Lenovo ThinkCentre M70s Tiny [26] was used. It has an Intel Core i5 processor running at 2.5 GHz, 8 GB of RAM, 1 RJ45 Ethernet port, 4 USB 2.0 and 4 USB 3.0 ports, Bluetooth 5.0, 1 HDMI 2.1 port, an HD SSD with a capacity of 256 GB, and runs the Windows 10 operating system.

An Apple MacBook Pro [27] was used. It features an Intel Core i7 6-core processor running at 2.6 GHz, 16 GB of RAM, a 512 GB HD SSD, four Thunderbolt 3 (USB-C) ports, Bluetooth 5.0, and Wi-Fi 802.11ac. macOS Monterey was the operating system.

Raspberry Pi [28] is equipped with a Broadcom BCM2711 processor, a quad-core Cortex-A72 (ARM v8) 64-bit processor running at 1.5 GHz, 8 GB of RAM, Bluetooth 5.0, 1 Gigabit Ethernet port, 2 USB 3.0 and 2 USB 2.0 ports, 40 general-purpose input/output (GPIO) pins, and a Micro SD card slot used for loading the Raspbian operating system (a Debian-based Linux distribution) and storing data.

The Odroid N2+ board [29] is powered by a quad-core Cortex-A73 (ARM v8) processor running at 2.4 GHz and has 4 GB of RAM. It is equipped with 1 RJ45 Ethernet port, 4 USB 3.0 ports, 1 Micro USB2.0 port, 1 HDMI 2.0 port, and a Micro SD card slot for the operating system and data storage. The operating system is Ubuntu.

The Jetson Nano [30] features a quad-core ARM A57 (ARM v8) processor running at 1.43 GHz, 4 GB of RAM, 1 RJ45 Ethernet port, 1 HDMI port, 1 display port, and 4 USB 3.0 and USB 2.0 Micro-B ports. Similar to the previous boards, it also has a Micro SD card slot, and the operating system is Linux4Tegra, based on Ubuntu 18.04.

Both Odroid and Jetson boards have dedicated fans that periodically turn on, increasing the power consumption over Raspberry.

Table 1 shows the main characteristics and differences of these elaboration units.

### 2.3. Proposed Pipeline

**Figure 4:**
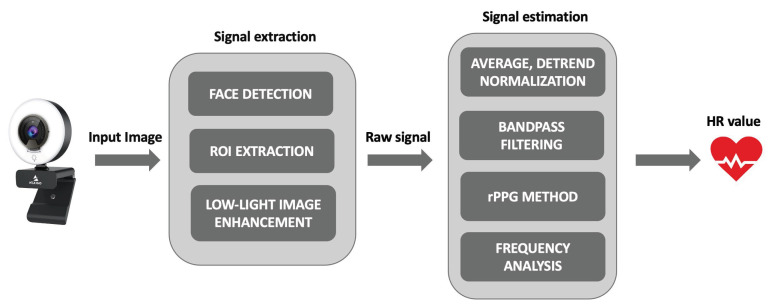
Graphical representation through a block diagram of the pipeline designed and implemented for HR estimation by a plethysmographic signal extracted from the face of an end-user.

A stream of images acquired from the commercial camera introduced in Section 2.1 served as the input for our proposed system. A graphical representation of the pipeline through logical blocks is depicted in Figure 4. The algorithmic blocks were designed and implemented with the goal of achieving accurate HR estimation while keeping processing time, power consumption, and computational load low, and considering a real-world environment as the acquisition context. For the latter point, algorithmic logic was designed to provide the measurement output (even without optimal ambient lighting conditions).

#### 2.3.1. Signal Extraction

**Figure 5:**
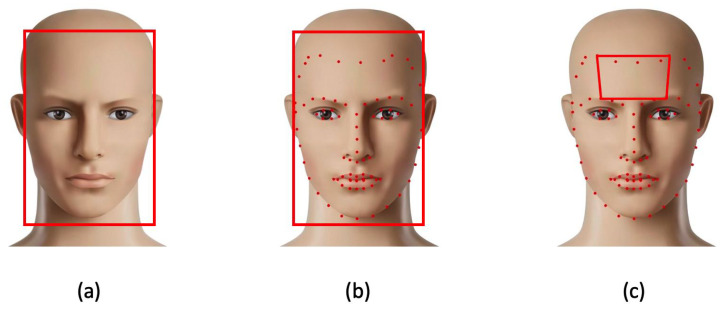
(**a**) Output of face detection step; (**b**) identification within the face of 81 landmarks (red dots); (**c**) ROI selection for rPPG signal extraction (the forehead) using a subset of the landmarks.

The first algorithmic step, which is preparatory to extracting the rPPG signal for HR estimation, involves identifying the subject’s face in the image and selecting salient regions of the face (ROIs) where a more pronounced PPG signal is present. For face detection and landmark extraction, we used Dlib’s 68-point facial landmark detector [31]. This widely used face detection model is based on the histogram of oriented gradients (HoG) features and Support Vector Machine (SVM). The model consists of five HOG filters: front-facing, left-facing, right-facing, front-facing but rotated left, and front-facing but rotated right. The dataset used for training consisted of 2825 images obtained from the (LFW) dataset [32]. The advantages of this methodology are as follows: (a) it is the fastest method on the CPU; (b) it is a lightweight model that works very well for frontal and slightly non-frontal faces; (c) it can detect faces and landmarks under small occlusions. However, the detector, in its original version, has the following disadvantages: (a) it does not work for side faces and extreme non-frontal faces, such as looking down or up; (b) the bounding box of the face often excludes part of the forehead or even part of the chin. To eliminate the latter critical issue, and considering that the scientific literature has shown that the forehead and cheeks have stronger rPPG signals than other areas of the face [13,33], an extended version of this library was utilized, which provides 13 additional landmarks that delineate the forehead (Figure 5).

Next, a low-light image enhancement algorithm was implemented to improve the brightness of the selected ROI (forehead region). The algorithm is designed to balance ROI brightness while preserving the details, such as color variations within the forehead that are related to blood volume. Unlike traditional histogram equalization methods, the algorithm uses a double automatic platform approach based on an upgraded Cuckoo Search (CS) algorithm [34]. First, the histogram obtained is segmented, and the platform limit is selected based on the histogram statistics and improved CS technology. The sub-histograms are clipped by two platforms, and histogram equalization is performed as required. This produces a forehead with nice contrast and balanced brightness. Following the application of these algorithmic blocks, the RGB values inside the ROI are much less disturbed by ambient lighting.

The video segment used for continuous monitoring of vital signs is represented by a sliding temporal window. It refers to the segment of the video from which we estimate the discrete value of HR. Sliding windows ranging from 10 to 60 s are generally used in the relevant literature to estimate vital signs. In our work, we adjusted the sliding window size to increase the algorithm’s sensitivity to physiological changes and make it more workable for real-time implementation. Consequently, to segment the raw RGB data, a window size of 30 s was used, with a 1 s slide between each set of windows.

#### 2.3.2. Signal Estimation

The raw RGB signals extracted from the forehead region are then processed by averaging the pixel values to generate temporal signals. Next, specific signal processing techniques are used to increase the signal quality for the following step of our proposed pipeline. First, detrending is applied for removing the linear trends from the raw signals and, subsequently, since the interest is in the periodicity of the signals, the resulting raw signals are then normalized by dividing them by their maximum absolute values and smoothing using a sliding average filter.

Another algorithmic step preparatory to rPPG signal extraction is the raw signal filtering operation, which aims to achieve a good signal-to-noise ratio and remove unrealistic frequencies. In this step, the raw RGB signals are passed through a third-order band-pass filter with ideal behavior, which removes components outside of the frequency band (0.7–4 Hz), which are typically used in the literature for HR estimation. This band corresponds to a HR range of 42–240 beats per minute (bpm).

Following that, the inputs to any rPPG method employed in the time domain for the extraction of the pulse signal and subsequent HR estimation are the three pre-processed temporal signals, obtained for each color band of the RGB space and expressed as R(t), G(t), and B(t), where t represents the instant within the time window at which the discrete values of R, G, and B (between 0 and 1 after normalization) are measured. Different methods for extracting the rPPG signal have been explored in the scientific literature in the recent past. For example, principal component analysis (PCA) [5] and ICA [35] are rPPG methods based on blind source separation (BSS), without supervision or data labeling. In these approaches, a significant rPPG signal was usually found in the second component. Moreover, the GREEN method [36] is a frequent rPPG method, which is based on the concept that of the three color channels, the green channel is the most similar to the PPG signal and can be used as its estimate.

All the mentioned methods have advantages and disadvantages. For example, BSS methods show limited success when the video streaming capture environment is not controlled. Moreover, PCA suffers from the presence of motion artifacts in original signals. Finally, the GREEN method has a limitation in assuming that the rPPG signal is contained exclusively in the green channel color. Following these considerations and evaluating the most recent results presented in the scientific literature, we implement the CHROM method [37] in our proposed approach for retrieving the rPPG signal. The chrominance signals are generated from the RGB traces with the use of a skin-tone standardized linear combination compatible with different skin colors. The CHROM method assumes that the light reflected from the skin generally occupies similar coordinates in the RGB space under white illumination.

From an algebraic perspective, the CHROM method starts with a zero standard deviation normalization. Next, the three color components R(t), G(t), and B(t) are projected into two orthogonal chrominance vectors, which are expressed through the following equations:

(1) $$\begin{array}{ccc} X_{CHROM}\left(t\right) & = & 3*R(t)-2*G(t) \end{array}$$

(2) $$\begin{array}{ccc} Y_{CHROM}\left(t\right) & = & 1.5*R(t)+G(t)-1.5*B(t) \end{array}$$

At this point, the rPPG signal is calculated by combining the obtained chrominance vectors by the following formula:

(3) $$rPPG\left(t\right)=X_{CHROM}\left(t\right)-\mu Y_{CHROM}\left(t\right)$$

where μ is equal to the ratio of the standard deviations of $X_{CHROM}\left(t\right)$ and $Y_{CHROM}\left(t\right)$.

The discrete value of HR can be estimated from the obtained rPPG signal in two ways: (1) peak detection and (2) frequency analysis. In the peak detection approach, individual peaks are used to extract HR, while in the frequency analysis approach (which is commonly adopted in the literature and used in this work), the elaborated rPPG signal is converted to the frequency domain using techniques such as Fast Fourier Transform (FFT). To estimate the HR value, the frequencies related to the peaks of the power spectrum are considered, specifically the frequency $f_{HR}$ corresponding to the maximum peak’s intensity in the frequency band (0.7–4 Hz). Finally, HR values, expressed as average bpm, are estimated by multiplying the frequency values $f_{HR}$ by 60.

## 3. Results and Discussion

The proposed approach was validated to verify its real-time operation by conducting a series of tests on the processing units described in Section 2.2. These tests were carried out at the "Smart Living Technologies" laboratory of the Institute for Microelectronics and Microsystems (IMM) in Lecce, Italy. Due to COVID-19 restrictions, the entire platform was tested using 10 colleagues from the Institute, with an average age of 46.13 ± 7.25. Before the experimentation stage, informed consent was obtained from all subjects involved.

Our experiments were conducted using the same software implemented in Python on the different elaboration units. The vision sensor described in Section 2.1 was used for the acquisition of the image stream. For each subject, 5 measurements were repeated at the following distances from the vision sensor: 0.5 m, 1.0 m, and 1.5 m. In addition, 3 different frame acquisition resolutions were considered: 320 × 240, 640 × 480, and 1280 × 720. The acquisition time was set to 30 s for each measurement session.

During the experimental phase, each individual wore a commercial pulse oximeter OXI-2 manufactured by GIMA [38] as the ground-truth to obtain the HR value to compare with the predicted values using our system.

The accuracy of the HR measurement method was evaluated using the percentage of cases for which the absolute error between the ground truth and the estimator falls below 3 bpm [39,40] as the metric, while varying the frame acquisition resolution and the distance from the vision sensor. The results achieved are reported in Table 2, which shows, first of all, the dependence of the pipeline’s accuracy on the distance between the subject’s face and the vision sensor, regardless of the considered elaboration unit. The obtained results indicate a high accuracy at distances of 0.5 m and at 1.0 m, with values always higher than 97% (except for Raspberry); a gradual deterioration is noted when the subject’s face is at a distance of about 1.5 m. The frame resolution also affects the accuracy; the results obtained in the case of 640 × 480 and 1280 × 720 resolution are quite similar. This allowed the use of a resolution of 640 × 480, thereby reducing the computing power required for processing the image itself. In order to verify the quality of the measurements, the ANOVA statistical method was used, which provides us with useful information regarding the correlation between the obtained measurements on the analyzed platforms at varying frame resolutions and distances. This indicates that the test of the relationship between the different platforms measuring HR is statistically acceptable. Table 2 shows the results of the ANOVA analysis, reporting the average *p*-values, from which it can be deduced that there is a high probability that the data obtained are reliable.

Then the benchmarking of the proposed pipeline on the described embedded platforms was analyzed due to its important impact on the usability of the application.

First, in order to assess the actual convenience of using ARM-based platforms as processing units, a comparative analysis was conducted by evaluating the average execution times of the algorithmic steps obtained on all platforms considered in this work. The proposed pipeline was evaluated according to the following three main steps: (1) face detection, (2) signal extraction, and (3) signal estimation. Table 3 shows the corresponding obtained average execution times. Raspberry had the highest total execution time compared to the other processing units due to the execution time of the block related to face detection as it did not have the GPU; this block used the Dlib library, which could significantly benefit from the use of a GPU. It is important to note that the other two ARM-based platforms achieved absolutely comparable runtimes with the PC and laptop. In particular, the laptop obtained the lowest execution times due to the hardware features, while Odroid N2+ and Jetson Nano had slightly lower execution times than the PC and not much higher than the laptop.

**Figure 6:**
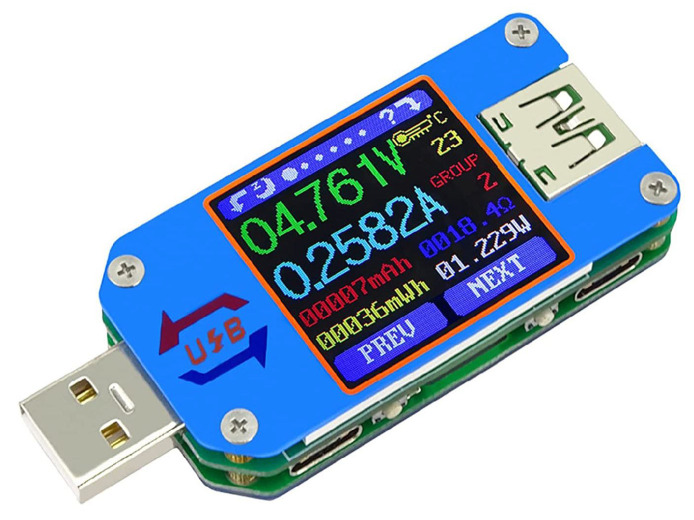
USB power meter tester used to estimate the power consumption.

Moreover, the efficiency of the proposed pipeline was evaluated by measuring the CPU load, memory usage, and power consumption on each considered hardware platform. To analyze the CPU load and memory usage, the Python library “psutil” was used; these values are reported in percentages. The power consumption of each ARM-based embedded platform was evaluated with a USB UM25C power meter [41] (Figure 6). It was possible to monitor the power consumption during the performed tests by using the iOS app [42].

As with the execution time, the CPU load, memory usage rate, and power consumption were evaluated by running 10 tests for each involved user. Then, the average of all performances was calculated for each user, generating 10 values for each parameter. Finally, the results were averaged, resulting in the average of the three benchmarking values.

**Figure 7:**
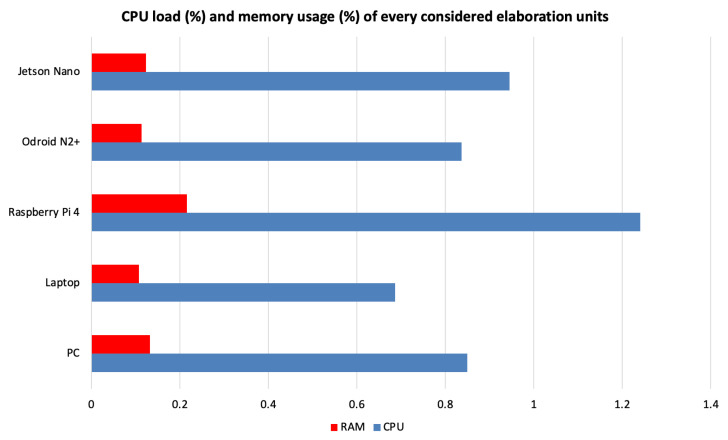
CPU load (%) and memory usage (%) of the five considered elaboration units.

Figure 7 shows the comparison of the CPU load and memory usage between the five analyzed platforms.

In terms of the CPU load, the trend shows a load with values between approximately 0.7% and 1.24% across platforms. Raspberry Pi 4 showed the highest load undoubtedly due to the absence of GPU, which is instead present on the other two ARM-based platforms, which, in fact, have a CPU load absolutely comparable to the PC and slightly lower than the laptop.

The trends in memory usage show fairly similar behavior on all the hardware platforms considered, with the exception of Raspberry, which shows slightly higher memory usage. However, the platform architecture does not seem to particularly influence memory usage. In fact, we can see that both Odroid N2+ and Jetson Nano have values similar to the PC and laptop with a range between 0.107% and 0.132% of their capacity, while Raspberry Pi 4 has a rate of 0.216%.

**Figure 8:**
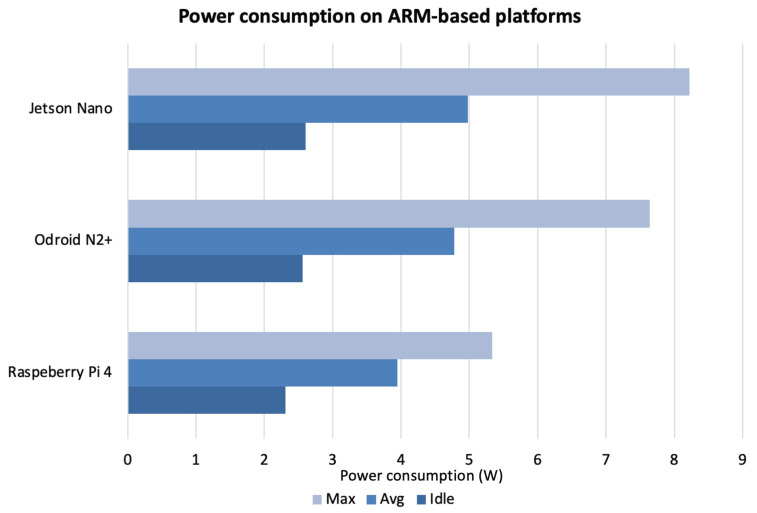
Idle, average, and maximum power consumption (W) in ARM-based embedded boards.

Regarding the power consumption on the ARM-based embedded boards, various external peripherals, such as the keyboard, mouse, and monitor, were integrated into boards, resulting in higher power consumption. The achieved results are shown in Figure 8, where the idle, average, and maximum power consumption for the three platforms considered are plotted.

Raspberry Pi 4 had the lowest power consumption, likely due to the lack of a cooling unit, which is present on the other two embedded platforms. In fact, both Odroid N2+ and Jetson Nano have higher power consumption. However, this higher power consumption compared to Raspberry does not seem to affect the CPU load and RAM usage, which are lower than on Raspberry. Thus, both the Odroid N2+ and Jetson Nano offer a good trade-off between computation time, CPU load, RAM usage, and power consumption.

**Figure 9:**
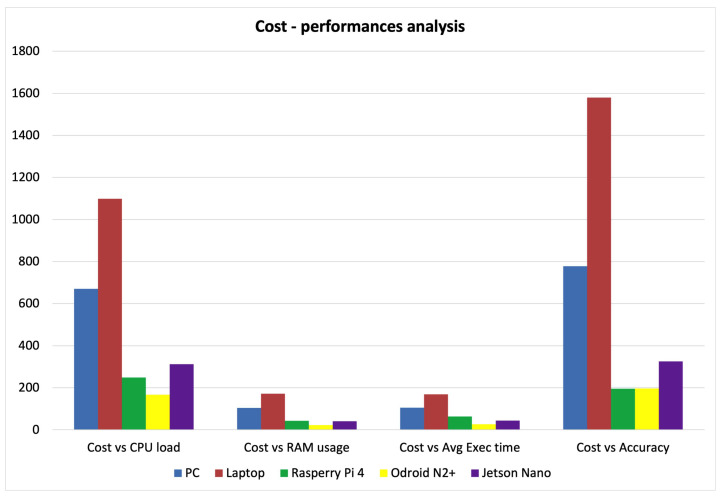
Cost-performance analysis on the considered elaboration units.

Finally, considering the possible use of the proposed architecture in an ambient-assisted living (AAL) environment, another parameter to take into account is the platform cost. To this end, the relationship between the costs of the various analyzed platforms and the performances used for the benchmark was investigated. In particular, the obtained values for the five processing units for the CPU load (a), RAM usage (b), average execution times (c), and pipeline accuracy (d), respectively, are plotted in Figure 9. In particular, accuracy was achieved for all platforms a resolution of 640 × 480 and at a distance of 0.5 m. As expected, ARM-based embedded boards have a significantly lower ratio, as their cost is about 3–4 times lower than that of the PC and about 5–8 times lower than that of the laptop. In particular, as can be observed, among the ARM-based platforms, Odroid N2+ performed the best considering its cost and good performance.

## 4. Conclusions

The COVID-19 pandemic has led to increased use of contactless technologies for monitoring vital signs. Patients with medical issues other than COVID-19 may not be required to visit hospitals, thereby reducing the risks of cross-infection. This feature will undoubtedly be favored by vulnerable groups, such as the elderly or those with pre-existing health conditions like cardiovascular and respiratory disorders, who may be reluctant to use sensors that typically fall into the wearable device category [43].

It is well known that cameras and webcams can be used as detectors in various monitoring scenarios, including activity recognition, human behavior understanding, and affective computing. Consequently, it is also advantageous to utilize vision sensors for contactless measurement of vital signs, taking advantage of their presence in living environments.

With the advancements in the Internet of Things (IoT) and big-data ML technologies in the past decade, continuous monitoring of human vital signs, such as HR, has become feasible in many scenarios, offering enormous potential for healthcare applications. The need to avoid travel and to stay at home during the pandemic period has motivated researchers and companies to develop solutions that perform well from the measurement accuracy point of view, but are also low cost. The objective of the present research work was two-fold: firstly, to design and implement an algorithmic pipeline for HR estimation through the use of the rPPG signal extracted from a specific facial ROI; and secondly, to test the real-time operation of the pipeline on five different hardware platforms, including a desktop PC, a laptop, and three embedded platforms. In addition, its operation was also against different operating systems.

Regarding the accuracy of HR measurement with respect to ground-truth, we conducted various experiments in a laboratory setup by changing the distance of the observed subject and the resolution of the input image. The objective was to validate the pipeline’s performance in contexts similar to the real environment. The results demonstrated the pipeline’s remarkable generalization capability across different environmental setups and processing platforms, with a deviation of less than 3 bpm between the real measurement and ground-truth. We also measured specific computational metrics, such as CPU load, RAM usage, and average execution time of algorithmic steps, to determine the best score in terms of the performance-to-market price ratio in the tested hardware platforms. Our experiments concluded that the Odroid N2+ embedded platform provided the best results. To our knowledge, there are no scientific publications in the literature comparing commercial embedded platforms for the development of an intelligent sensory node for contactless HR monitoring.

Some limitations of the present work should also be highlighted. First, the experimentation took place under controlled laboratory conditions, so the effectiveness of some algorithmic steps cannot be generalized. For example, the algorithmic module that regulates ambient lighting may not work properly in environments where the influence of natural light is greater. Another limitation lies in the number and age of end-users involved in the tests. As a future development, first, the whole system (algorithmic pipeline plus commercial hardware) should be tested in real environments. In addition, it will be necessary to verify that the algorithmic choices made are also valid in the case of monitoring elderly subjects (to obtain valid technology in the AAL context). Finally, it would be useful to verify the performances of other remote photoplethysmographic algorithms (e.g., with deep learning approaches or by adding additional blocks to the proposed pipeline) on the considered ARM-based platforms.

## Figures and Tables

**Table 1:** Comparison of the PC, Laptop, and ARM-based-embedded units.

Hardware	PC	Laptop	Raspberry	Odroid	Jetson
Model	Lenovo ThinkCentre M70s Tiny	Apple MacBook Pro	Pi 4 Model B	N2+	Nano
CPU	Intel Core i5	Intel Core i7	Quad Core ARM Cortex-A72	Quad Core ARM Cortex-A73	Quad Core ARM A57
RAM	8 GB	16 GB	8 GB	4 GB	4 GB
Connectivity	Bluetooth, Wifi, Ethernet, USB	Bluetooth, Wifi, USB-C	Bluetooth, Wifi, Ethernet, USB	Bluetooth with adapter, Wifi, Ethernet, USB	Bluetooth with adapter, Wifi, Ethernet, USB
Video output	HDMI	-	mini HDMI	HDMI	HDMI, display port
Storage	256 GB SSD	512 GB SSD	32 GB SD-Card	32 GB SD-Card	32 GB SD-Card
Dimensions	W. 340 mm D. 298 mm H. 92.5 mm	W. 349 mm D. 241 mm H. 15.5 mm	W. 88 mm D. 58 mm H. 19.5 mm	W. 90 mm D. 90 mm H. 17 mm	W. 100 mm D. 80 mm H. 29 mm
Weight	5200 g	1830 g	46 g	200 g	250 g
Energy consumption	180–200 W	87 W	2–6 W	2.2–6.2 W	5–10 W
Operating Voltage	AC 220V DC 19V	AC 240V DC 20V	AC 220V DC 5V	AC 220V DC 12V	AC 220V DC 5V
Operating system	Windows 10	Monterey	Raspbian	Ubuntu	Linux4Tegra
Cost	€789	€1599	€200	€199	€330

**Table 2:** Average accuracies and average *p*-values of the HR estimation at varying distances from the vision sensor and frame resolution.

		320 × 240		640 × 480		1280 × 720	
		Accuracy	*p*-Value	Accuracy	*p*-Value	Accuracy	*p*-Value
PC	0.5 m	97.2	0.581	98.6	0.752	98.8	0.758
	1 m	95.8	0.424	97.3	0.599	97.5	0.638
	1.5 m	94.5	0.327	96.4	0.495	96.8	0.543
Laptop	0.5 m	97.6	0.657	98.8	0.758	98.9	0.779
	1 m	96.1	0.448	97.5	0.638	97.6	0.657
	1.5 m	94.9	0.380	96.8	0.543	97.1	0.562
Raspberry	0.5 m	95.2	0.409	97.6	0.657	97.7	0.676
	1 m	94.7	0.354	95.9	0.435	96.1	0.448
	1.5 m	93.6	0.197	94.8	0.363	95.0	0.397
Odroid	0.5 m	97.4	0.619	98.6	0.752	98.8	0.758
	1 m	95.9	0.435	97.2	0.581	97.3	0.599
	1.5 m	94.3	0.298	96.2	0.462	96.6	0.511
Jetson	0.5 m	97.4	0.619	98.7	0.755	98.8	0.758
	1 m	95.9	0.435	97.2	0.581	97.3	0.599
	1.5 m	94.3	0.298	96.3	0.477	96.7	0.528

**Table 3:** Average execution times (sec) for each pipeline step.

	PC	Laptop	Raspberry Pi 4	Odroid N2+	Jetson Nano
Face detection	0.0910	0.0723	0.2542	0.0913	0.0908
Signal extraction	0.0048	0.0037	0.0079	0.0044	0.0043
Signal estimation	0.0370	0.0297	0.0524	0.0368	0.0369
Total time	0.1328	0.1057	0.3145	0.1325	0.1320

## Data Availability

The data presented in this study are available upon request from the corresponding author. The data are not publicly available due to restrictions, as they contain information that could compromise the privacy of research participants.
